# HARTMANN PROCEDURE OR RESECTION WITH PRIMARY ANASTOMOSIS FOR TREATMENT OF PERFORATED DIVERTICULITIS? SYSTEMATIC REVIEW AND META-ANALYSIS

**DOI:** 10.1590/0102-672020200003e1546

**Published:** 2021-01-15

**Authors:** Rogério Perônico BEZERRA, Adriano Carneiro da COSTA, Fernando SANTA-CRUZ, Álvaro A. B. FERRAZ

**Affiliations:** 1 Department of Surgery, Federal University of Pernambuco, Recife, PE, Brazil

**Keywords:** Acute diverticulitis, Colorectal surgery, Colectomy, Postoperative complications, Diverticulite aguda, Cirurgia colorretal, Colectomia, Complicações pós-operatórias

## Abstract

**Background:**

The Hartmann procedure remains the treatment of choice for most surgeons for the urgent surgical treatment of perforated diverticulitis; however, it is associated with high rates of ostomy non-reversion and postoperative morbidity.

**Aim:**

To study the results after the Hartmann vs. resection with primary anastomosis, with or without ileostomy, for the treatment of perforated diverticulitis with purulent or fecal peritonitis (Hinchey grade III or IV), and to compare the advantages between the two forms of treatment.

**Method:**

Systematic search in the literature of observational and randomized articles comparing resection with primary anastomosis vs. Hartmann’s procedure in the emergency treatment of perforated diverticulitis. Analyze as primary outcomes the mortality after the emergency operation and the general morbidity after it. As secondary outcomes, severe morbidity after emergency surgery, rates of non-reversion of the ostomy, general and severe morbidity after reversion.

**Results:**

There were no significant differences between surgical procedures for mortality, general morbidity and severe morbidity. However, the differences were statistically significant, favoring primary anastomosis in comparison with the Hartmann procedure in the outcome rates of stoma non-reversion, general morbidity and severe morbidity after reversion.

**Conclusion:**

Primary anastomosis is a good alternative to the Hartmann procedure, with no increase in mortality and morbidity, and with better results in the operation for intestinal transit reconstruction.

## Graphical Abstract

[Fig f1a]

**Figure f1a:**
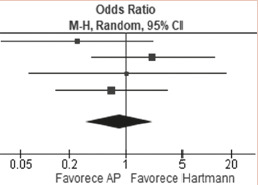
Mortality after emergency surgery in randomized controlled trials

## RESUMO

**Racional:**

O procedimento a Hartmann permanece sendo o tratamento de escolha da maioria dos cirurgiões para o tratamento cirúrgico de urgência da diverticulite perfurada, entretanto está associado com altas taxas de não reversão da ostomia e de morbidade pós-operatória.

**Objetivo:**

Estudar os resultados após o procedimento de Hartmann vs. ressecção com anastomose primária, com ou sem ileostomia, para o tratamento da diverticulite perfurada com peritonite purulenta ou fecal (grau de Hinchey III ou IV), e comparar as vantagens entre as duas formas de tratamento.

**Método:**

Busca sistemática na literatura de artigos observacionais e randomizados comparando ressecção com anastomose primária vs. procedimento de Hartmann no tratamento de urgência da diverticulite perfurada. Analisar como desfechos primários a mortalidade após a operação de urgência e a morbidade geral após ela; como desfechos secundários, a morbidade severa após a operação de urgência, as taxas de não reversão da ostomia, a morbidade geral e severa após a reversão.

**Resultados:**

Não houve diferenças significativas entre os procedimentos cirúrgicos para mortalidade, morbidade geral e morbidade severa. Contudo, as diferenças foram significativas estatisticamente favorecendo anastomose primária na comparação com procedimento de Hartmann nos desfechos taxas de não reversão do estoma, morbidade geral e morbidade severa após reversão.

**Conclusão:**

A anastomose primária apresenta-se como boa alternativa ao procedimento de Hartmann, sem aumento de mortalidade e morbidade, e com melhores resultados na operação de reconstrução do trânsito intestinal.

## Graphical Abstract

[Fig f1as]

**Figure f1as:**
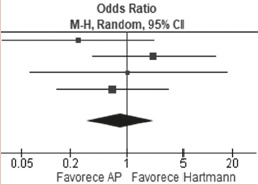
Mortalidade após operação de urgência nos ensaios clínicos randomizados

## INTRODUCTION

Diverticular disease is a common gastrointestinal disease and found in one third of people over 60 in the Western world^4^. One of its main complications is diverticulitis, and it can be classified as uncomplicated (Hinchey classification I and II), and complicated (Hinchey classification III and IV)^9^. About 25% of patients with acute diverticulitis require emergency intervention, and the standardized treatment for the perforated form with fecal or purulent peritonitis (Hinchey III and IV classification) is emergency surgery^4^ ^,^ ^24^.

Hartmann’s procedure (PH) - which consists of resection with construction of terminal colostomy - remains the preferred option for most surgeons. However, several studies suggest that resection with primary anastomosis (AP) is the same as the Hartmann procedure in terms of postoperative mortality and morbidity^11^.

The objective of this systematic review with meta-analysis was to study the results after the Hartmann vs. resection with primary anastomosis, with or without ileostomy, for the treatment of perforated diverticulitis with purulent or fecal peritonitis (Hinchey grade III or IV), and to compare the advantages between the two forms of treatment, through the evaluation of mortality, post-morbidity surgery and ostomy non-reversion rates.

## METHODS

The Scopus, Medline/Pubmed, Web of Science, SpringerLink, Elsevier, PMC, Wiley Online Library databases were consulted through the CAPES journals portal, and searches were carried out on the Cochrane Library and Embase databases. For the research, the terms “diverticulitis”, “primary anastomosis”, “Hartmann’s procedure” were used combined through the Boolean operator ‘AND’. No date or language filters have been added. Additionally, an individual search was made for articles cited in the identified works that were relevant to the study. This systematic review was developed based on the Cochrane Manual for systematic reviews of interventions (Cochrane Handbook for Systematic Reviews of Interventions) and on PRISMA (checklist and flow chart of selection of articles). The question to be answered by the research was structured based on the acronym PICO: (P) patients included were adults over 18, who underwent emergency surgical treatment for perforated diverticulitis of the left colon; (I) analyzed intervention was resection with primary anastomosis (AP) with or without protective ostomy; (C) the primary anastomosis would be compared to the Hartmann procedure; (O) the results compared would be mortality and morbidity in urgent and reversal operations, in addition to the rate of non-reversion of the ostomy.

### Eligibility criteria and outcomes

This review included observational studies and randomized clinical trials, which were divided for the purpose of analyzing results into two subgroups, one containing observational studies (subgroup 1) and the other randomized clinical trials (subgroup 2).

### Inclusion and exclusion criteria

Group 1 included observational articles and clinical trials comparing resection with primary anastomosis, with or without protective ostomy, and the Hartmann procedure for the surgical treatment of perforated left colon diverticulitis in patients over 18 years of age who underwent emergency surgery. Articles that did not compare the two techniques, or that included elective procedures and other causes of colon perforation that were not due to diverticulitis were excluded. In subgroup 2, articles with the same previous criteria were included, and articles that included patients with intraoperative findings compatible with grades I and II of the Hinchey classification were excluded.

### Primary outcomes

Primary outcomes were assessed individually in the two subgroups, with overall mortality and morbidity being analyzed after the emergency operation. Events that occurred within the first 30 days after surgery were included in general mortality and morbidity. 

### Secondary outcomes

The secondary outcomes evaluated were severe morbidity after the emergency operation, general morbidity after stoma reversal, severe morbidity after reversal and non-reversion rate of the ostomy. These outcomes were studied only in subgroup 2. Severe morbidity was defined as a complication with a degree greater than or equal to IIIb of the classification of Clavien-Dindo’s surgical complication^13^.

### Data collection and analysis

The studies found were analyzed by two researchers (RPB and ACC) independently and were selected based on the inclusion and exclusion criteria. The differences regarding the inclusion or not of a certain article were discussed with a third researcher (AABF), in order to reach consensus.

The data collected included author, year of publication, length of follow-up, Hinchey degrees, number of patients undergoing each intervention, postoperative mortality, general morbidity after emergency and reversal procedures, severe postoperative morbidity, severe morbidity after reversal, and ostomy non-reversion rates.

### Bias risk analysis

Observational (subgroup 1) and randomized (subgroup 2) articles were evaluated in separate meta-analyzes to reduce the risk of bias. Randomized clinical trials were individually assessed using the Cochrane tool for risk of bias, which assesses randomization, allocation secrecy, blinding scheme, intention-to-treat analysis.

### Statistical analysis

The following variables were evaluated after the emergency operation: general mortality; general morbidity; severe morbidity; general morbidity and stoma reversal; severe morbidity after reversal; and rate of non-reversion of the ostomy. All variables are dichotomous, and the odds ratio (OR) was chosen to measure the corresponding effect. Predicting possible heterogeneity between the included studies, the random effect model was used, and since the studies had small sample sizes and events, the Mantel-Haenszel method with a 95% confidence interval (CI) was used. P=0.05 was considered statistically significant. The heterogeneity between studies for each outcome was measured using the chi-square test and the Higgins inconsistency test (I^2^). The results of the meta-analysis were presented in the form of a forest plot. The statistical program used for the meta-analysis calculations was Review Manager 5.3 (RevMan).

## RESULTS

**FIGURE 1:**
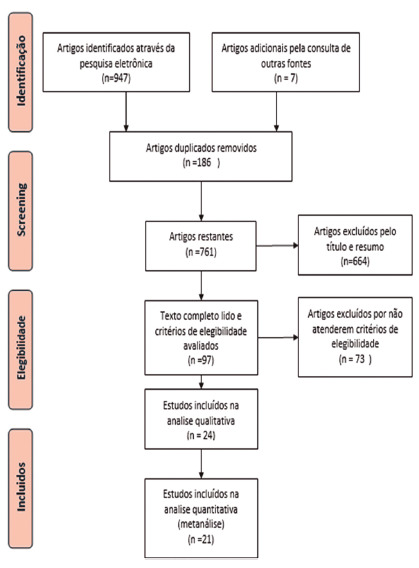
Identification and selection of articles

The electronic search strategy resulted in the identification of 947 articles; of these, 186 were repeated. Of the remaining 761, 664 were excluded by reading the title and summary, as they related to other subjects, such as laparoscopic lavage, damage control, fistulas, diseases other than diverticulitis, did not compare the two interventions or were not observational clinical studies or randomized. There were 97 articles left that were read in full, among these 73 did not meet the eligibility criteria, and were excluded, which resulted in 24 articles selected for qualitative analysis, of which four were randomized clinical trials; of these, 21 were assessed qualitatively and quantitatively by meta-analysis. Figure 1 shows PRISMA flowchart for the search strategy.

### Mortality after emergency surgery in observational studies (Subgroup 1)

Most of the studies included in this analysis did not show statistically significant differences between primary anastomosis with or without a protective ostomy and the Hartmann procedure, despite the tendency towards lower mortality rates with primary anastomosis^1^ ^,^ ^3^ ^,^ ^5^ ^,^ ^8^ ^,^ ^17^ ^,^ ^19^ ^,^ ^20^ ^,^ ^21^ ^,^ ^26^ ^,^ ^27^. In five studies, lower mortality rates for primary anastomosis were observed, with a statistically significant difference^7^ ^,^ ^12^ ^,^ ^14^ ^,^ ^18^ ^,^ ^22^; however, these studies showed statistically significant differences between the preoperative and intraoperative characteristics of patients in the variables comorbidities, ASA, degree of Hinchey, Mannhein Peritonitis Index (Table 1). Only one study^23^ showed higher mortality for patients undergoing AP compared to Hartmann; however, as in this study there was a small number of patients (n=8) with purulent or fecal peritonitis undergoing AP, the effects of events could be overestimated. To avoid this problem, studies were excluded from the meta-analysis in which less than 10 patients were submitted to one of the compared procedures, thus avoiding overestimation of these events and reducing the heterogeneity between studies.

**TABLE 1:** Study characteristics and differences between AP and PH interventions in each study

	Year	Type	Patients	Intervention		Patient characteristics that were statistically different between the two groups in each study
				AP	PH	
Alizai^1^	2013	NR	Hinchey I to IV	26	72	Hinchey II, III and IV, MPI
Breitenstein^3^	2007	NR	Hinchey II to IV*	30	30	No differences
Capasso^5^	2003	NR	Hinchey III to IV	19	19	No report
Gawlick^7^	2012	NR	Hinchey I to IV	340	678	No differences
Gooszen^8^	2001	NR	Hinchey I to IV*	32	28	No differences
Hold^10^	1990	NR	Hinchey III and IV	16	31	No report
Lee^12^	2019	NR	Hinchey I to IV	208	2521	Mean age, ASA>III and comorbidities
Mueller^14^	2011	NR	Hinchey I to IV*	47	26	Hinchey III/IV, comorbidities, ASA IV
Regenet^17^	2003	NR	Hinchey III and IV	27	33	No differences
Richter^18^	2006	NR	Hinchey III and IV	36	5	MPI
Schilling^19^	2001	NR	Hinchey III and IV	13	42	No differences
Sileri^20^	2014	NR	Hinchey III and IV	48	40	No differences
Thaler^21^	2000	NR	Hinchey III and IV	20	62	ASA IV/V, MPI
Trenti^22^	2011	NR	Hinchey I to IV*	27	60	Mean age, ASA, Hinchey III/IV
Tudor^23^	1994	NR	Hinchey III and IV	8	44	No report
Wedell^26^	1997	NR	Hinchey III and IV	14	15	No report
Zingg^27^	2010	NR	Hinchey I to IV	46	65	Mean age, ASA, Hinchey, CCI, MPI
Binda^2^	2012	R	Hinchey III and IV	34	56	No differences
Lambrichts^11^	2019	R	Hinchey III and IV	64	66	No differences
Bridoux^1^	2017	R	Hinchey III and IV	50	52	No differences
Oberkofler^15^	2012	R	Hinchey III and IV	32	30	No differences

AP=resection with primary anastomosis; PH=Hartmann’s procedure; NR=not randomized; R=randomized

**FIGURE 2:**
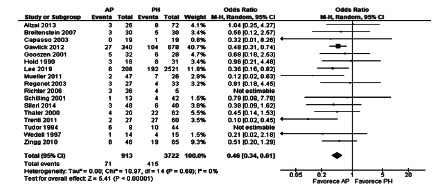
Forest plot of mortality after emergency surgery in observational studies

**FIGURE 3:**
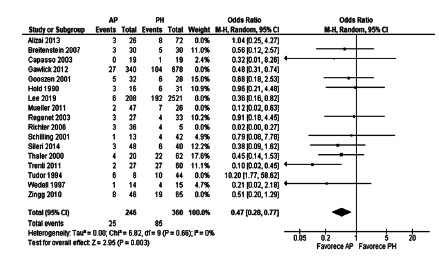
Forest plot of mortality after emergency operation of observational studies with only Hinchey III and IV patients

The meta-analysis of mortality of all observational articles (subgroup 1) demonstrated that AP has a lower mortality rate when compared to PH, this difference being statistically significant (OR 0.46, [CI: 0.34-0.61], p<0.001). The heterogeneity by the Chi-square method was 10.97 and the I^2^=0% (Figure 2). When only studies with data from Hinchey III and IV patients were analyzed to reduce possible selection biases, AP had lower mortality (OR 0.45, [0.27-0.76], p=0.003, Figure 3).

### General morbidity after emergency surgery: observational studies (Subgroup 1)

Of the included observational studies, 12 presented data on general morbidity^1^ ^,^ ^3^ ^,^ ^5^ ^,^ ^8^ ^,^ ^12^ ^,^ ^14^ ^,^ ^17^ ^,^ ^19^ ^,^ ^20^ ^,^ ^21^ ^,^ ^22^ ^,^ ^27^, among these nine did not present statistically significant differences in morbidity between AP and PH^1^ ^,^ ^5^ ^,^ ^8^ ^,^ ^12^ ^,^ ^14^ ^,^ ^19^ ^,^ ^21^, and three lower rates of general morbidity for patients undergoing AP, this difference being significant statistically (p=0.05) ^17,20,22^.

**FIGURE 4:**
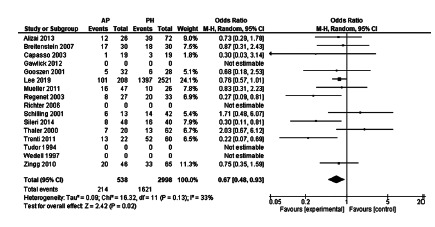
General morbidity after emergency surgery in observational studies

The meta-analysis of general morbidity after emergency surgery showed a significant difference in favor of AP (OR=0.67, [CI: 0.48-0.93], p=0.02). The calculation of heterogeneity resulted in Chi^2^=16.32 and I^2^=33% (Figure 4).

### Mortality after emergency surgery: randomized clinical studies (Subgroup 2)

In this review, four randomized clinical trials^2,4,11,15^ were included, and none of them showed statistically significant differences in postoperative mortality when resection with primary anastomosis and the Hartmann procedure were compared.

**FIGURE 5:**
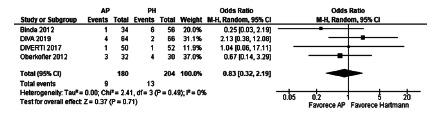
Mortality after emergency surgery in randomized controlled trials

The meta-analysis of the mortality results of these articles did not demonstrate statistically significant differences between the two surgical procedures under analysis (OR 0.83, [0.32-2.19], p=0.71. The heterogeneity was Chi^2^=2.41 and I^2^=0% (Figure 5).

### General morbidity after emergency surgery: randomized clinical trials (Subgroup-2)

Randomized clinical trials did not show significant differences in relation to postoperative morbidity, when resection with primary anastomosis and the Hartmann procedure were compared.

**FIGURE 6:**
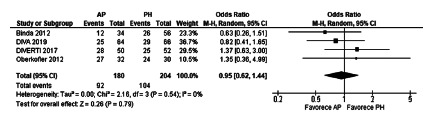
General morbidity after emergency surgery in randomized controlled trials

The meta-analysis of general morbidity in the first 30 postoperative days did not show statistically significant differences between the two surgical procedures under analysis (OR 0.95, [0.62-1.44], p=0.79). The heterogeneity was Chi^2^=2.16 and I^2^=0% (Figure 6).

### Severe morbidity after emergency surgery: randomized clinical studies (Subgroup-2)

Severe morbidity was defined by the Clavien-Dindo classification as greater than or equal to IIIb. Among the randomized clinical trials, none showed significant differences in relation to severe morbidity after emergency surgery.

**FIGURE 7:**
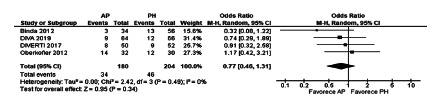
Severe morbidity after emergency surgery in randomized controlled trials

The meta-analysis of severe morbidity in the first 30 postoperative days did not show statistically significant differences (OR 0.77, [0.43-1.31], p=0.34). The heterogeneity was Chi^2^=2.42 and I^2^=0% (Figure 7).

### Analysis of ostomy non-reversion rates

Among the randomized clinical trials, two did not present significant differences between the rates of ostomy non-reversion, despite the favorable results to AP^2^ ^,^ ^4^. The other two^9,11^ had statistical significance when comparing the rates of non-reversion between AP and PH, with the rates of ostomy reversal, being higher in resection with primary anastomosis and protective ostomy

**FIGURE 8:**
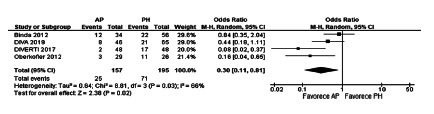
Rate of non-reversion of the ostomy

In the meta-analysis of the four studies, a lower rate of non-reversion of the ostomy was found among patients undergoing AP, this difference being statistically significant (OR=0.30, [0.11-0.81], p=0.002). The heterogeneity was Chi^2^=8.81 and I^2^=66% (Figure 8).

### General morbidity after ostomy reversal operation

Among the randomized clinical trials, two did not present significant differences in general morbidity after the ostomy reversal operation, despite the favorable results to AP^11^ ^,^ ^15^. The other two randomized clinical trials showed statistical significance when comparing general morbidity after reversion, with a lower incidence of complications after reversal of ostomies performed to protect the primary anastomosis, when compared to complications of reversal of the PH ostomy^2^ ^,^ ^4^.

**FIGURE 9:**
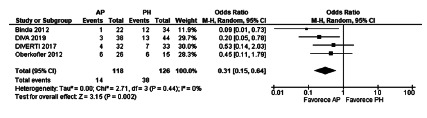
General morbidity after a reversal operation

In the meta-analysis of the four studies, a lower rate of general complications was found after the ostomy reversal among patients undergoing AP, with this difference being statistically significant (OR=0.31, [0.15-0.64], p=0.002. The heterogeneity was Chi^2^=2.71 and I^2^=0% (Figure 9).

### Severe morbidity after ostomy reversal operation

**FIGURE 10:**
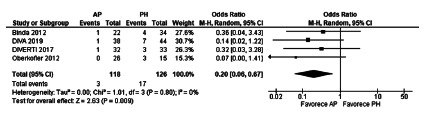
Severe morbidity after reversal in randomized controlled trials

Although none of the articles alone showed significant differences in the rates of serious complications after the ostomy reversal, the meta-analysis demonstrated that the ostomy reversal performed to protect the primary anastomosis has lower rates of severe morbidity when compared with the reversal of the PH ostomy, this difference being statistically significant (OR=0.20, [0.06-0.67], p=0.009, Figure 10).

### Clinical significance

In the subgroup 1 meta-analysis, statistically significant differences were found for the postoperative mortality outcome, with lower rates among patients undergoing resection with primary anastomosis with or without protective ostomy, when compared with those submitted to the PH (OR 0.46, [CI: 0.34-0.61], p<0.001). Likewise, the analysis of post-surgical general morbidity in subgroup 1 revealed better results in patients submitted to AP with statistical significance (OR=0.67, [CI: 0.48-0.93], p=0.02). In contrast, subgroup 2 meta-analysis showed no differences in mortality (OR 0.83, [0.32-2.19], p=0.71), general morbidity (OR 0.95, [0.62-1 , 44], p=0.79), and severe morbidity after emergency surgery (OR 0.77, [0.43-1.31], p=0.34). However, the differences were statistically significant, favoring AP compared to PH in the following outcomes: stoma non-reversion rates (OR=0.30, [0.11-0.81], p=0.002); general morbidity after reversal (OR=0.31, [0.15-0.64], p=0.002) and severe morbidity after reversal (OR=0.20, [0.06-0.67], p=0.009).

### Sensitivity analysis and publication bias

**FIGURE 11:**
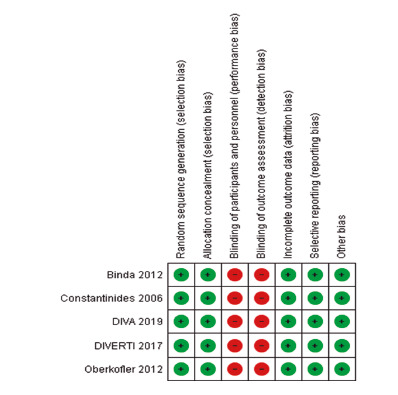
Summary of the risk of bias attributed to each randomized clinical trial according to the authors’ judgment

**FIGURE 12:**
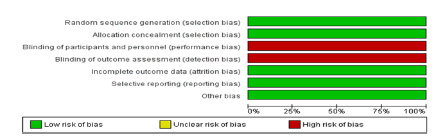
Graph with percentage representation of the risk of bias in each study according to the authors’ judgment

**FIGURE 13:**
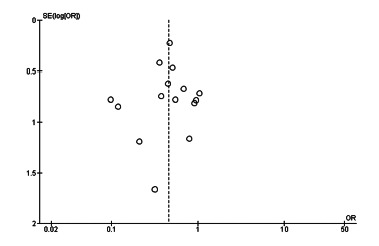
Funnel plot of mortality after emergency surgery in subgroup 1

To increase the sensitivity of the research, randomized clinical trials were analyzed separately from the other articles included, as they had a higher level of evidence, and were not subject to the selection bias of observational studies (Figures 11 and 12). In addition, within the analysis of observational studies, meta-analyzes were performed with all articles, and another only with articles that included patients Hinchey III and IV or reported these data separately. Studies that had a total number of participants less than 10 in one arm were excluded from the meta-analysis of the outcome in question. The analysis of the risk of publication bias in subgroup 1 was performed using a funnel plot for mortality (Figure 13). To avoid the risk of publication bias of randomized clinical trials, a rigorous search for articles related to the topic was carried out, and only four articles were found.

## DISCUSSION

The Hartmann procedure has been the choice for most surgeons in the emergency for the treatment of perforated diverticulitis, despite being associated with high rates of stoma non-reversion, which can reach 50%, and high postoperative morbidity^1^ ^,^ ^3^ ^,^ ^6^ ^,^ ^7^ ^,^ ^8^ ^,^ ^22^ ^,^ ^25^. The justification for its use is the prerogative that primary anastomosis in the context of purulent or fecal peritonitis would be more prone to anastomosis dehiscences, thus increasing the mortality rates and morbidity of the emergency operation^2^ ^,^ ^4^ ^,^ ^11^ ^,^ ^12^ ^,^ ^15^ ^,^ ^16^ ^,^ ^22^.

Observational studies (subgroup 1) when individually evaluated did not show increased mortality and morbidity when resection with primary anastomosis, with or without protective ostomy, was used in comparison to the PH in the emergency for perforated diverticulitis^1^ ^,^ ^3^ ^,^ ^5^ ^,^ ^8^ ^,^ ^17^ ^,^ ^19^ ^,^ ^20^ ^,^ ^21^ ^,^ ^26^ ^,^ ^27^. It was possible to evidence a trend towards better mortality and morbidity rates after resection with primary anastomosis. In four of the included studies, this trend was statistically significant^7^ ^,^ ^12^ ^,^ ^14^ ^,^ ^18^ ^,^ ^22^. In assessing the combined form through meta-analysis, these studies demonstrated lower rates of mortality and morbidity when AP was used, when all studies were included, as well as when only observational studies with Hinchey III and IV patients were analyzed.

In view of the above results, resection with AP with or without the making of a protective ostomy proved to be a good alternative to the PH in the treatment of complicated diverticulitis, and presents similar or even better rates of mortality and morbidity after resection, but with higher stoma reversal rates^2^ ^,^ ^3^ ^,^ ^4^ ^,^ ^8^ ^,^ ^11^ ^,^ ^15^ ^,^ ^16^ ^,^ ^25^. However, in observational studies, the choice of the type of surgical procedure performed is the responsibility of the surgeon, and this choice is often based on scores that assess the general condition of the patient and locoregional factors of the disease, but with a tendency to perform the PH for patients with worse clinical conditions. This fact generates a selection bias for the most severe patients, and consequently with greater propensity for postoperative mortality and morbidity included in the Hartmann group, and for those with more favorable characteristics submitted to AP, with statistically significant differences between the two groups (Table 1), thus having an impact on surgical results. Thus, the best results of resection with primary anastomosis may be the result of this bias, suggesting the performance of randomized clinical trials to evaluate the best surgical procedure for perforated diverticulitis.

In subgroup 2, randomized clinical trials were evaluated, four of which were identified after an exhaustive search^2^ ^,^ ^4^ ^,^ ^10^ ^,^ ^11^. In these studies, the decision of the surgical treatment to be used in each patient was made by randomization, thus eliminating the selection bias present in observational studies and, consequently, in these studies, patients undergoing AP and PH were statistically comparable in terms of their demographic characteristics, comorbidities and locoregional characteristics of the disease.

The meta-analysis of mortality and general morbidity in subgroup 2, despite the tendency towards better results for AP, did not reveal statistically significant differences, in contrast to the meta-analysis of these outcomes in subgroup 1, where these differences were significant. This fact confirms the hypothesis that the differences found in subgroup 1 are due to differences in the distribution of patients between procedures; however, more randomized studies should be performed to elucidate these outcomes. However, it can be said that AP can be an option to PH in perforated diverticulitis without increasing mortality and general morbidity in the emergency room.

Severe morbidity, defined as Clavien-Dindo greater than or equal to IIIb in the first 30 postoperative days, was assessed by meta-analysis in subgroup 2 and did not show significant differences between AP and PH, it is important to note that anastomosis dehiscences with need of reoperations in AP are among the factors causing severe morbidity in patients undergoing this procedure. Despite the absence of these dehiscences in patients undergoing PH, other complications of similar severity occurred in this surgical procedure, resulting in similar severe morbidities between the two groups with a tendency to better results with AP. In the subgroup 2 meta-analysis, the outcomes of stoma non-reversion rates, general morbidity after reversal and severe morbidity after reversal in the differences, were statistically significant favoring AP over PH.

For even better elucidation of the presented outcomes, more randomized studies should be carried out on the topic so that they can be included in future systematic reviews like this one

## CONCLUSION

Resection with primary anastomosis can be used as an alternative to the Hartmann procedure in patients undergoing urgent surgery for perforated diverticulitis, without increasing mortality, general morbidity and severe morbidity after the resection operation. It has advantages in ostomy reversal rates and in general and severe morbidity after this procedure.
